# Treatment Outcomes of Revascularization and Cervical Pulpotomy in Immature Traumatized Maxillary Incisors: A Case Report With 24 Months Follow‐Up

**DOI:** 10.1002/ccr3.71244

**Published:** 2025-10-12

**Authors:** Motahareh Khosrojerdi, Mana Mowji, Mohammadhossein Sadeghi

**Affiliations:** ^1^ Pediatric Dentistry Department Faculty of Dentistry, Mashhad University of Medical Sciences Mashhad Iran; ^2^ Community Oral Health Department Faculty of Dentistry, Mashhad University of Medical Sciences Mashhad Iran

**Keywords:** calcium‐enriched mixture, cervical pulpotomy, dental trauma, immature tooth, regenerative endodontics, vital pulp therapy

## Abstract

This report compares regenerative endodontic procedures (REPs) and vital pulp therapies (VPTs) in traumatized immature maxillary incisors of a 7‐year‐old patient. Following a bicycle accident, teeth #9 and #8 presented with crown fractures, subluxation, and negative pulp tests. Tooth #9, which had undergone discoloration, was treated 13 days after dental trauma using a revascularization procedure with a calcium‐enriched mixture of cement, whereas tooth #8 underwent cervical pulpotomy with zinc oxide‐eugenol after 43 days. Both were restored with composite resin. After 24 months, both teeth remained symptom‐free, vital, and showed continued root development; tooth #9 achieved a complete dentinal bridge and apical closure, and tooth #8 exhibited apical narrowing and dentinal‐wall thickening. Both methods were effective, but revascularization resulted in faster and more substantial root maturation. The findings of this study indicate that both treatment modalities, when applied within their respective indications, are effective options with a high likelihood of success.

**Summary**

- The clinical and radiographic findings of this study indicate that, although current practice emphasizes conservative vital pulp therapies for immature permanent teeth, regenerative treatments can also serve as effective options in appropriate cases.
- These approaches, including cervical pulpotomy, demonstrate a high likelihood of success when used according to their specific indications.

## 1 Introduction

Several protocols are available for treating immature permanent teeth with open apices and pulp necrosis. When part of the pulp remains viable, vital pulp therapy (VPT) is preferred in the literature: necrotic tissue is removed, the residual healthy pulp is preserved, and root development can continue [1]. Clinically, VPT encompasses stepwise caries removal, indirect or direct pulp capping, and miniature, partial, or full pulpotomy, selected according to the volume of coronal pulp that can be retained [2]. Over time, a range of materials has been utilized for pulp capping, each with distinct advantages and limitations. Calcium hydroxide (CH) has historically served as the gold standard for this procedure. This status is primarily attributed to its clinical effectiveness and straightforward application. However, CH is associated with several limitations, including poor long‐term sealing ability, potential for dissolution over time, and formation of tunnel defects [3]. Recent materials, such as mineral trioxide aggregate (MTA), calcium‐enriched mixture (CEM), and Biodentine, have received increased attention due to their reported performance [4, 5]. CEM cement can stimulate early reparative dentine formation and increase pulp cell activity. These properties suggest CEM may serve as an alternative to CH [5].

Extensive crown fractures can limit the available space for partial pulpotomy and placement of coronal restorative materials. If there is a significant delay between the traumatic event and treatment initiation, cervical pulpotomy is indicated [6].

Regenerative endodontic procedures (REPs) as an alternative treatment for pulp management in immature teeth comprise apexification and revascularization [7]. REPs are commonly chosen when the pulp is completely necrotic. Apexification traditionally relies on either long‐term CH dressings [8] or only placement of a MTA apical plug [9] to create a calcified barrier. Although both techniques effectively create an artificial apical barrier, they share the drawback of preventing further root development or dentinal‐wall thickening [10]. Introduced later, revascularization offers a regenerative approach that supports continued root development and canal‐wall reinforcement, providing natural apexogenesis rather than an artificial barrier [11].

This report presents a dual‐treatment approach in a 7‐year‐old boy, applying revascularization to a completely necrotic immature incisor and a cervical pulpotomy to a partially necrotic counterpart.

## 2 Case History/Examination

The report was prepared in accordance with the 2020 Preferred Reporting Items for Case Reports in Endodontics (PRICE) guidelines. All procedures were carried out in the Pediatric Dentistry Department of Mashhad Dental School. The study complied with the ethical standards set forth in the Declaration of Helsinki, and written consent for both treatment documentation and image publication was secured from the patient's legal guardian.

**FIGURE 1:**
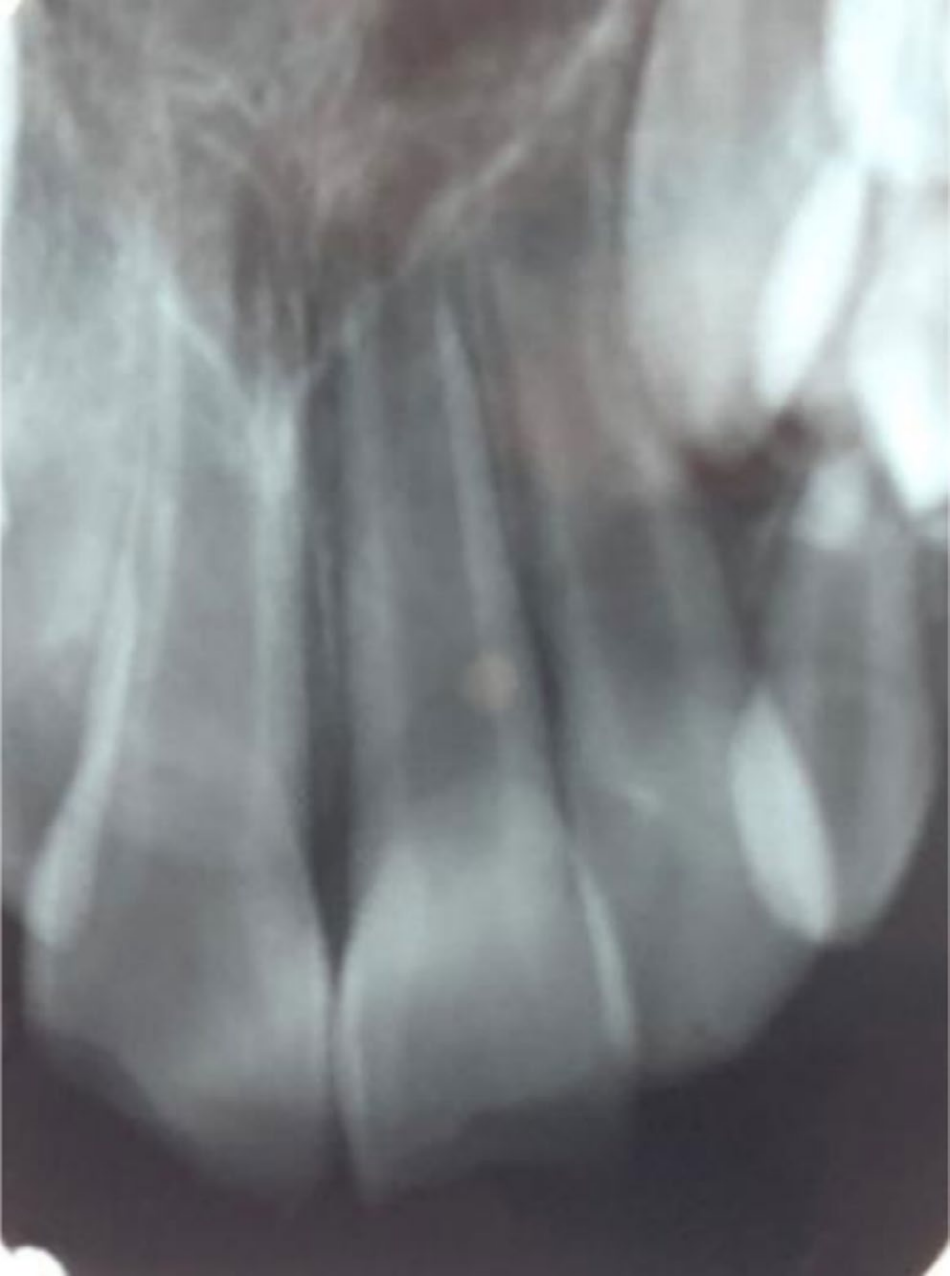
Preoperative radiograph.

A 7‐year‐old boy attended the Pediatric Clinic of Mashhad Dental School complaining of gray‐to‐pink discoloration of tooth #9. He had sustained a bicycle accident 13 days earlier (13 May 2023), resulting in uncomplicated crown fractures and mild subluxation of both maxillary central incisors. Preoperative radiograph is shown in Figure 1. Unfortunately, due to the high volume of patients in the pediatric dental emergency department on the day of the patient's visit, initial photographs could not be obtained.

## 3 Methods (Differential Diagnosis, Investigations and Treatment)

At the first visit, the patient exhibited no extraoral swelling or soft‐tissue injury. Intraoral examination revealed uncomplicated enamel‐dentine fractures of both maxillary central incisors. Tooth #9 appeared gray, was slightly mobile (< 1 mm), and tender to percussion; tooth #8 presented a similar fracture line with comparable tenderness and mobility. Thermal (cold spray) and electric pulp tests were negative for both teeth. Periapical radiographs confirmed immature roots with open apices (≈5 mm in diameter for both teeth) and slight periodontal‐ligament widening, with no evidence of root fracture. Computed‐tomography imaging was not pursued because of cost constraints.

**FIGURE 2:**
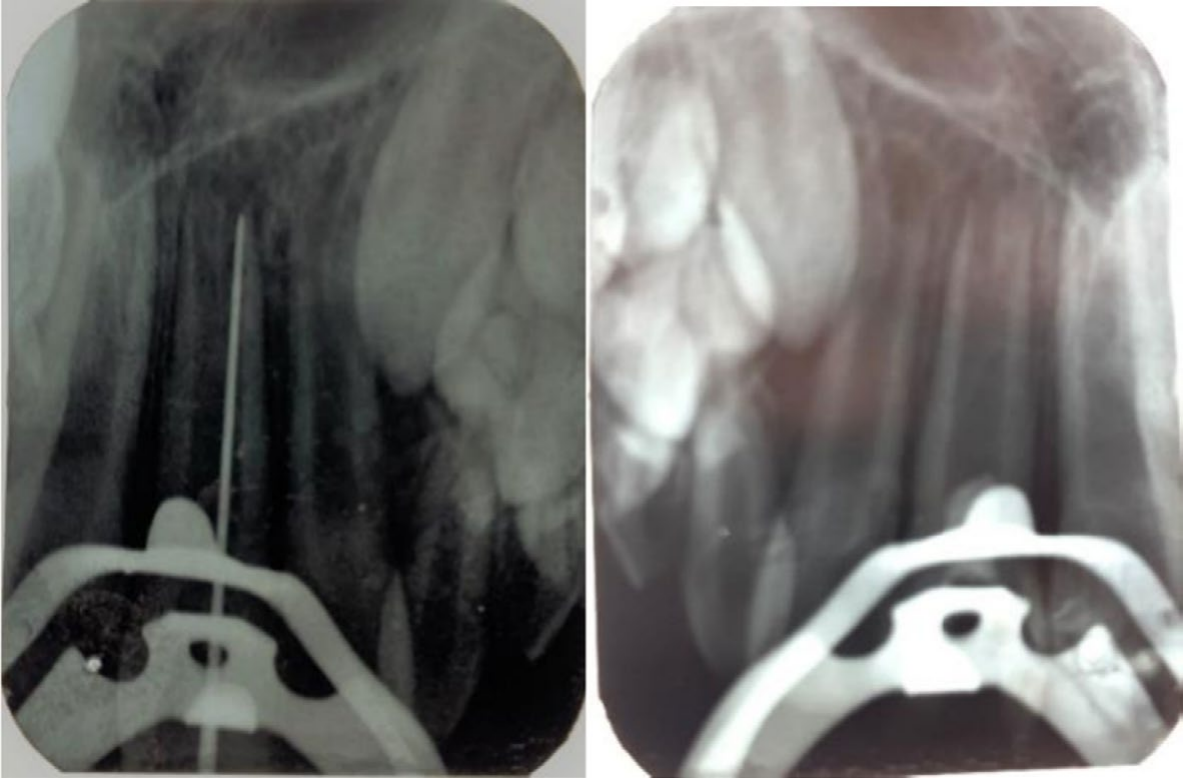
Radiographs of revascularization procedures.

Because pulp necrosis was strongly suspected in tooth #9 (negative vitality tests and gray discoloration), a cavity test was performed without local anesthesia. The patient remained pain‐free during access preparation, and a #35 K‐file introduced to 19 mm from the incisal edge revealed no bleeding, confirming complete necrosis (Figure 2). Given the necrotic pulp and the 5 mm apical foramen diameter, a regenerative protocol was initiated for this tooth.

**FIGURE 3:**
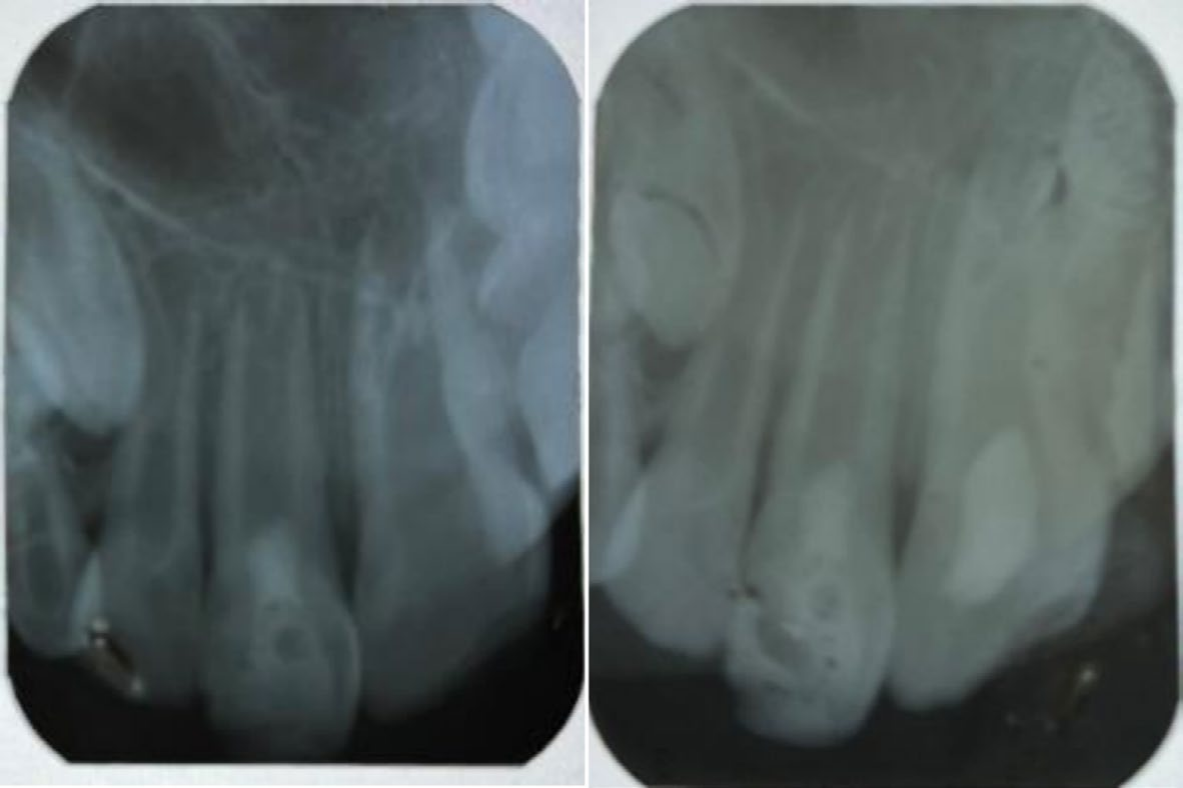
Radiographs of cervical pulpotomy procedures (1 month after initial visit).

**FIGURE 4:**
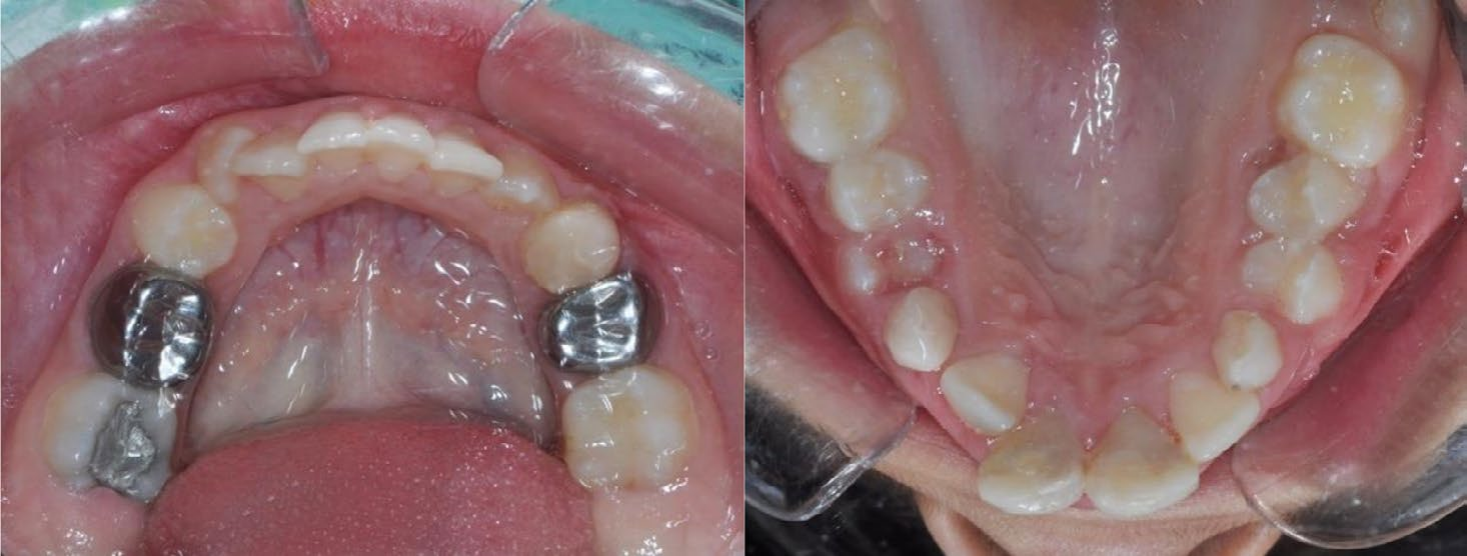
Occlusal view of the maxilla and mandible at the two‐year follow‐up.

For tooth #8, transient apical periodontitis was considered in the differential diagnosis; therefore, the tooth was monitored without intervention at the first visit. However, at the one‐month review, the patient reported spontaneous pain and increased percussion sensitivity in tooth #8. Clinical examination of tooth #8 revealed spontaneous pain, increased sensitivity to percussion, negative sensitivity tests, and apical rarefaction (Figure 3). These clinical indicators led to an initial diagnosis of pulp necrosis, prompting preparation of an access cavity. Subsequent irrigation and removal of necrotic tissue from the coronal pulp chamber exposed vital pulp tissue in the radicular region. Because necrosis was limited to the coronal area, the diagnosis was revised to partial pulp necrosis, and cervical pulpotomy was performed. After the treatment of the traumatized teeth, all carious lesions in the patient's other teeth were also managed (Figure 4).

### 3.1 Therapeutic Intervention—Tooth #9 (Revascularization)

Apart from the use of CEM cement instead of MTA, the revascularization protocol followed American Association of Endodontists (AAE) recommendations [12]. Under rubber‐dam isolation, the canal was gently irrigated with 20 mL of 1.5% sodium hypochlorite (Parcan, Septodont) using a side‐vented needle, dried, and filled with non‐setting CH paste (AH Temp, Dentsply Sirona). The access was sealed with Cavit (Coltozol, Aria Dent, Iran). After two weeks, the paste was removed, and the canal was flushed with 20 mL of 17% EDTA (MD Cleanser, Meta Biomed) for 5 min. After drying the canal with paper points, apical bleeding was induced with a pre‐bent size 40 K‐file to create a blood clot up to 2–3 mm below the cementoenamel junction. A 3 mm plug of CEM cement (Bionique Dent, Tehran) was placed over the clot, covered with light‐cured glass‐ionomer cement (Fuji II, GC), and the access was permanently restored with a universal adhesive and nanohybrid composite resin (Filtek Z350 XT, 3M ESPE).

### 3.2 Therapeutic Intervention—Tooth #8 (Cervical Pulpotomy)

After local anesthesia (2% lidocaine with 1:80,000 epinephrine; Xylocaine, Dentsply) and rubber‐dam isolation, the access cavity was prepared with a sterile diamond bur. Following the removal of necrotic coronal pulp tissue and achievement of hemostasis, the healthy radicular pulp was treated using a cotton pellet moistened with 5.25% sodium hypochlorite. Due to the unavailability of calcium silicate‐based cements such as mineral trioxide aggregate, the pulp stump was capped with a 2 mm layer of zinc oxide‐eugenol cement (IRM, Dentsply; powder by Kemdent, UK) and subsequently covered with zinc‐phosphate cement (Aria Dent, Iran). The tooth was immediately restored with the same adhesive/composite system used for tooth #9.

## 4 Conclusion and Results (Outcome and Follow‐Up)

The clinical outcomes of the treatments during follow‐up were evaluated using cold sensitivity tests, electric pulp testing (EPT), percussion sensitivity, discoloration, and radiographic signs.

**FIGURE 5:**
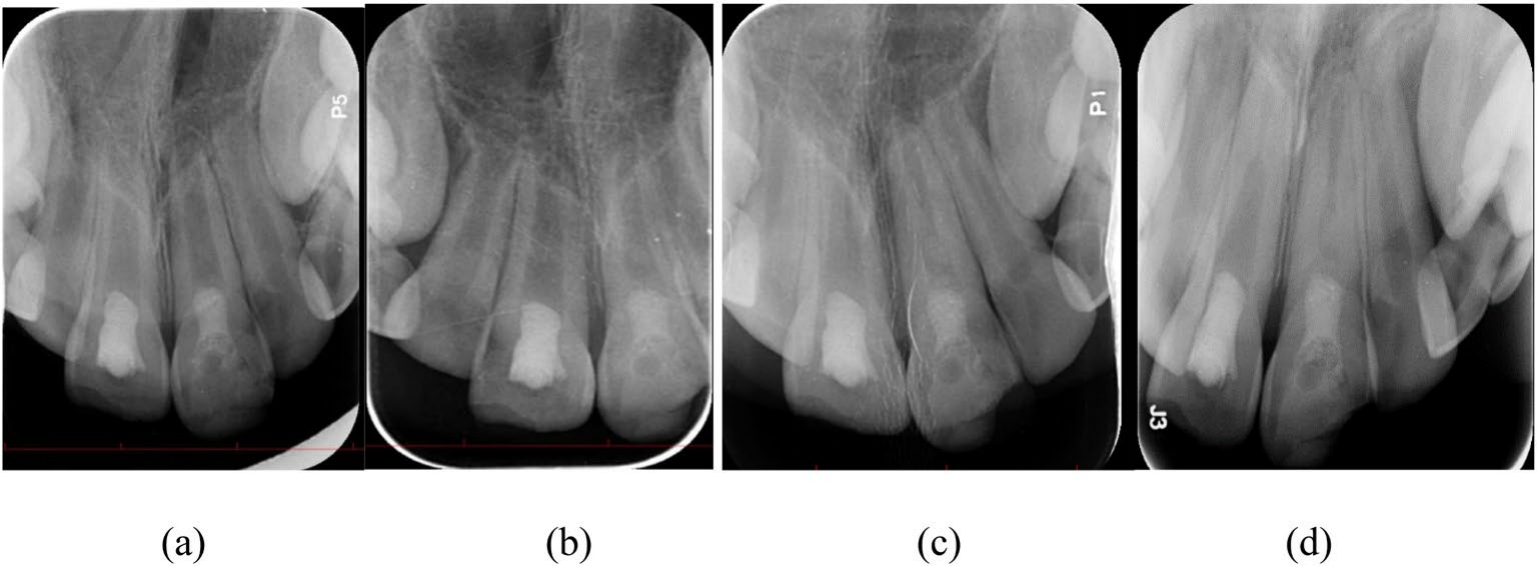
Follow‐up radiographs at (a) 3 months, (b) 6 months, (c) 12 months, and (d) 24 months.

**FIGURE 6:**
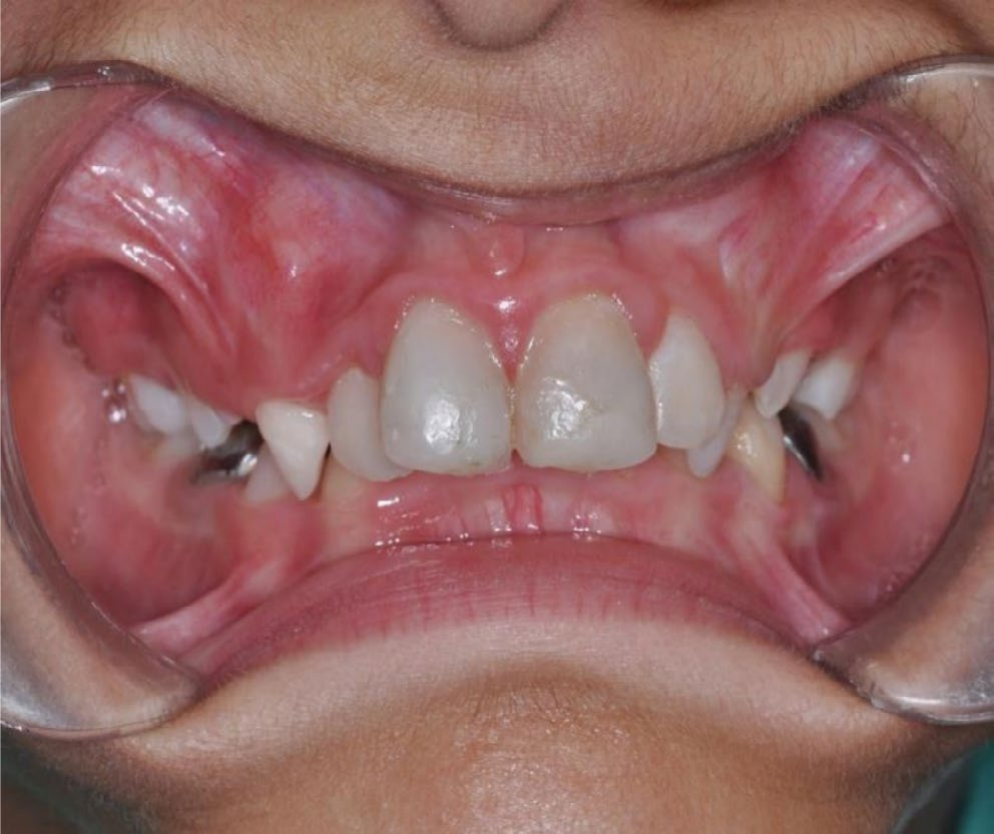
Clinical appearance at 24 months follow‐up.

Tooth #9 was treated with revascularization and calcium‐enriched cement treatment. This intervention resulted in progressive apical narrowing, significant thickening of the dentinal walls, and an increase in root length. Clinical assessments at 3, 6, and 12 months demonstrated uneventful healing, as shown in Figure 5. The tooth remained asymptomatic, responded normally to percussion, and exhibited no radiolucency or discoloration after the 6‐month follow‐up. At 24 months, long‐term stability was confirmed. The tooth did not respond to electric pulp testing or cold stimuli at this stage. The absence of response may be attributed to the formation of a thick dentinal bridge beneath the CEM barrier, which could have impeded the stimulus from reaching the pulp, as illustrated in Figure 5. Figure 6 presents the clinical appearance at 24 months, with tooth #9 displaying mild discoloration.

Tooth #8 underwent cervical pulpotomy with zinc oxide‐eugenol, resulting in complete dentine bridge formation and physiological apical closure. Clinical evaluations at 3, 6, and 12 months demonstrated uneventful healing, as shown in Figure 5. The tooth remained asymptomatic, responded normally to percussion, and showed no radiolucency or discoloration after 6 months. At 24 months, long‐term stability was observed. The tooth did not respond to electric pulp testing but remained sensitive to cold stimulation, indicating the presence of vital pulp tissue. Figure 6 shows the clinical appearance at 24 months, with tooth #8 exhibiting no discoloration.

To quantify structural changes, the baseline and 24‐month periapical radiographs were imported into Adobe Photoshop 2024 (Adobe Inc., San Jose, CA, USA). Horizontal and vertical magnification factors were calculated by measuring the known mesiodistal width of each crown on both images. After scaling the follow‐up film accordingly, matched reference points (cementoenamel junction, root apex, and mid‐root walls) were measured with the “Ruler” tool to the nearest 0.01 mm. Percentage change in root length and dentinal‐wall thickness was then derived for each tooth. The revascularized incisor (#9) showed a 64% overall gain in dentinal‐wall thickness and an 18% increase in root length, whereas the pulpotomized tooth (#8) displayed a 16% thickening in the cervical third, an 83% thickening in the apical third, and a 10% elongation of the root.

## 5 Discussion

Deep carious lesions and dental trauma can irreversibly injure the pulp in immature permanent teeth, leading to loss of vitality and cessation of root development [13]. For such teeth, therapy therefore centres on preserving a vital pulp to permit ongoing apexogenesis [14, 15]. Current debate suggests that VPT may now be favored over conventional root canal treatment not only in pediatric cases with open apices but also in mature permanent teeth [16]. In immature dentitions, the most reliable indicator of VPT success is radiographic evidence of continued root elongation and definitive apical closure [17].

In the present case, the maxillary right central incisor underwent a ZOE pulpotomy, whereas the left central incisor received revascularization, and both were immediately restored with composite resin. Two years later, clinical and radiographic assessments confirmed successful root development in both teeth; notably, the revascularized incisor exhibited more rapid maturation than its pulpotomized counterpart.

After correction for radiographic magnification, the revascularized incisor (#9) showed a 64% increase in overall dentinal‐wall thickness and an 18% gain in root length, whereas the ZOE‐pulpotomized tooth (#8) thickened by 16% cervically and 83% apically, with a 10% increase in length. The modest cervical apposition in the ZOE‐treated tooth may be explained by an unrecognized low‐grade coronal pulp inflammation present before treatment or by the eugenol component of ZOE provoking mild chronic irritation in the adjacent pulp tissue; either mechanism could have limited dentine deposition near the cervical area while allowing more robust formation toward the apex.

Calcium hydroxide has long been the traditional agent of choice for VPT in permanent teeth [13, 18]. Although the calcium ions released from Ca(OH)_2_ can stimulate reparative dentinogenesis at the exposure site [19], laboratory studies show that Ca(OH)_2_ is also cytotoxic and irritative to living pulp tissue [20]. These calcium ions, which are positively charged at physiological pH, bind to the negatively charged dentine surface and generate a calcium‐rich layer [21]. More recent work has reported high success rates for pulpotomies performed with MTA [4] and CEM cement [5], each offering comparable sealing ability and biocompatibility as pulp capping agents [16]. CEM cement is a bioactive hydraulic material with handling advantages, shorter setting time, and lower cost than MTA; its proven performance in regenerative procedures justified its use as the coronal barrier [22, 23].

ZOE, meanwhile, remains widely used for pulpotomy in primary molars [24]. Recognizing its potential in immature permanent teeth, several investigations have compared ZOE with MTA and found both materials clinically and radiographically successful, some favoring MTA [25], others reporting superior results for ZOE under optimal conditions [26, 27]. It also contains eugenol, which has analgesic and antimicrobial properties [28]. Considering CEM and MTA challenges of use in anterior teeth, like discoloration, ZOE was selected as the capping material in this case; furthermore, when subsequent root canal therapy is required for restorative reasons, ZOE's low risk of canal obliteration and reliable short‐term performance make it a practical choice [17].

In regenerative endodontics, canal disinfection is typically achieved with either triple antibiotic paste or CH. Once the canal is cleaned and disinfected, bleeding is deliberately induced from the periapical tissues; the resulting blood clot supplies both stem cells and a natural scaffold for tissue regeneration. Evidence suggests that, although platelet‐rich fibrin (PRF) and platelet‐rich plasma (PRP) can aid healing, the clinical and radiographic favorable outcomes of autologous blood clot are comparable with PRP and PRF [29]. Therefore, an additional scaffold was unnecessary in this case because streamlining the procedure improved the child's cooperation.

Several published reports echo the outcomes seen in our dual‐modality case. Sabbagh et al. [2] conducted cervical pulpotomy on an immature permanent molar with irreversible pulpitis and found that, after 50 months, the tooth remained functional and asymptomatic. Radiographic evaluation showed complete root development and a normal periodontal ligament, consistent with the findings of the present study. Despite damage to the periodontal tissues and Hertwig's epithelial root sheath during traumatic incidents, Cheng et al. concluded in a retrospective study that regenerative treatment procedures for necrotic teeth following trauma can yield favorable outcomes. The prognosis of regenerative therapy may depend on the type of traumatic injury and the extent of damage to the periodontal tissues and Hertwig's sheath [30]. Nevertheless, to date, systematic reviews have not demonstrated a significant difference in the success rates of regenerative treatments between teeth with necrosis caused by trauma and those with necrosis resulting from caries [31].

As well as Harandi et al. [17], who achieved apex closure with ZOE, MTA, and CEM but noted slight PDL widening in the ZOE tooth, our ZOE pulpotomy preserved vitality and promoted root completion, yet without the periodontal changes they observed. On the regenerative side, Dastpak et al. [32] reported a 12‐month follow‐up of a revascularisation case that showed both thickening of the dentinal walls and healing of the apical lesion, outcomes that closely resemble those observed in the present case. Similarly, the mature‐tooth REP described by Saad et al. [7] demonstrated foramen narrowing and intracanal hard‐tissue formation, aligning with the accelerated root maturation pattern we recorded in the immature incisor.

Systematic evidence reinforces these individual parallels. The metaanalysis by Pereira et al. [33] found no clear superiority between revascularisation and apexification in necrotic immature teeth, yet our side‐by‐side observation suggests that revascularisation can outperform VPT in speed of root development. Randomized trial syntheses by Glynis et al. [34] and Li et al. [35] reported overall REP success rates of ~95% in both immature and mature teeth, a figure consistent with our symptom‐free, radiographically sound outcomes at six months. Finally, Asgary et al. [13] concluded that pulpotomies performed with MTA or CEM generally outperform those using CH; however, our favorable ZOE outcome suggests that, given careful case selection and an adequate coronal seal, traditional materials can still deliver reliable results, consistent with earlier reports that found superior outcomes when ZOE is used correctly [26, 27].

Published follow‐ups vary widely, from 6 to 26 months, and tooth responses differ accordingly [36, 37]. As the literature suggests, longer monitoring, ideally 12–24 months, would better reveal additional root growth [12]; although shorter follow‐ups can limit the generalizability of the data [7]. Our report provides 24 months of observation, meeting the recommended interval, and is, to our knowledge, the first to compare revascularization and pulpotomy side by side in a single patient. Even so, larger clinical investigations with CBCT‐based follow‐ups are required to confirm and extend these findings.

This single‐case report cannot be generalized and lacks statistical power. Using different biomaterials—CEM for revascularization and ZOE for pulpotomy—introduces biological variability because of their distinct regenerative properties. Without CBCT imaging, it was not possible to perform a 3D assessment of root development and apical closure. No histologic confirmation was obtained; pulp vitality was only inferred from clinical and radiographic findings.

At 24 months, tooth #9 showed no response to EPT or cold tests. This may result from dentinal insulation under the CEM layer, but without advanced imaging or histology, this remains speculative. Tooth #9 exhibited mild discoloration, likely due to CEM, which created an aesthetic concern not addressed in this report. Emergency conditions prevented taking initial clinical photographs, limiting baseline visual documentation.

## 6 Conclusion

Protocol‐driven, pulp‐specific care can successfully manage traumatized immature incisors. Both the cervical pulpotomy and the revascularisation protocol produced symptom‐free teeth with radiographic healing. The findings of this study indicate that both treatment modalities, when applied within their respective indications, are effective options with a high likelihood of success.

## Author Contributions

**Motahareh Khosrojerdi:** conceptualization, investigation, writing – review and editing. **Mana Mowji:** investigation, writing – original draft. **Mohammadhossein Sadeghi:** investigation, writing – review and editing.

## Ethics Statement

The authors have nothing to report.

## Consent

A written consent form was obtained from the patient's parents for radiographs and other clinical information to be reported in the journal. The patient's parents understood that the patient's name and initial would not be published.

## Conflicts of Interest

The authors declare no conflicts of interest.

## Data Availability

The authors have nothing to report.
